# Editorial: Wired for Life

**DOI:** 10.3389/fmicb.2016.00662

**Published:** 2016-05-10

**Authors:** Amelia-Elena Rotaru, Pravin M. Shrestha

**Affiliations:** ^1^Department of Biology, Nordic Center for Earth Evolution, University of Southern DenmarkOdense, Denmark; ^2^Energy Biosciences Institute, University of California, BerkeleyBerkeley, CA, USA

**Keywords:** extracellular electron transfer, electrogens, electrotrophs, iron-reducers

Fossil fuels and fossil fuel derived chemicals are limited, non-renewable, and foreseen to run out in the near future. To find viable solutions for the production of renewable fuels and chemicals, significant efforts are being made by the scientific community. For example, some microorganisms have been shown to produce fuels or chemicals using renewable electricity charged electrodes as electron donors for their metabolism (Tremblay and Zhang). The ability to utilize insoluble substrates such as electrodes is due to the property of certain microorganisms to release or take up electrons from insoluble substrates, a process known as extracellular electron transfer (EET).

During EET to a solid electron acceptor, cells dispose of excess electrons indirectly by exploiting shuttles, or directly via a network of conductive pili and cytochromes (Lovley et al., 2011). The same EET mechanisms are considered to assist microorganisms when taking up electrons from solid electron donors, however less is known about this process (Tremblay and Zhang). Microorganisms also use direct EET for syntrophic interspecies interactions between a donor species and an acceptor species (Kouzuma et al.; Shrestha and Rotaru).

The papers in this research topic present insights into EET and how this process governs microbe–microbe, microbe–mineral, and microbe–electrodes interactions.

## Interactions between microbes and electrodes or minerals

### Isolation of novel bacteria capable of EET

In this research topic, three independent studies used unusual strategies to enrich or isolate novel bacteria capable of EET.

One study used an electrochemical strategy to isolate electrode and mineral-oxidizing bacteria (Rowe et al.). In this study, Rowe et al., used a three-step approach to enrich electro-active microorganisms from Catalina harbor sediments. Ultimately using solid substrates, like Fe^0^, FeS, and S^0^, 16 new species were isolated. All isolates were electroactive and related to six different genera, two of which were for the first time shown to include microorganisms capable of EET.

Li and Nealson studied microorganisms from marine sediments, which were able to use electrodes as electron acceptors. It is well known that sulfide accumulation inhibits the growth of many microorganisms, however conveniently sulfide can be removed electrochemically (Rabaey et al., 2006; Gong et al., 2013). By investigating electrode-associated microbial communities before and after electrochemical sulfide oxidation Li and Nealson were able to demonstrate a shift from primarily sulfate reducing species to a *Clostridia*- and *Arcobacter*-dominated system.

Novel EET microorganisms were also isolated by Hori et al. using iron-hydro-oxide minerals as electron acceptors. Six dissimilar Fe^3+^-reducers were isolated from different environments using acetate as electron donor. As a result, Hori et al. added five additional species to the known mineral-reducing genus *Geobacter* and one to *Pelobacter*.

### Real-time monitoring of microbial activity

Typically, when electrodes are used as terminal electron acceptor, microorganisms build biofilms on its solid surface. One paper in this research topic deals with real-time analyses of such electrode-associated biofilms. Aracic et al. highlighted real time gene-expression profiling, as method of choice, to assess the activity of electroactive biofilms.

Another paper by Wardman et al. showed how microbial activity can be monitored in real time in aquatic sediments using a simple anode-resistor-cathode device. Current production rates correlated with rates of anaerobic microbial activity more specifically with 2-^14^C-acetate turnover. The authors suggested to use this simple sensor technology for *in situ* monitoring of microbial activity during organic decontamination in sediments.

## Interspecies interactions

EET is also important for syntrophic interactions in natural or manmade methanogenic environments. Usually, syntrophic interactions are based on transfer of diffusible chemicals like H_2_ from a bacterium to a methanogenic archaeon. In a review, Kouzuma et al. highlighted, most recent discoveries in cooperative interactions including interactions via biotic and abiotic electrical conduits.

Many methanogenic archaea involved in syntrophy are hydrogenotrophs, scavenging excess reducing equivalents released, as H_2_, by their partner bacteria. However, methanogens from the family *Methanosarcinales* are also acetotrophs, and some feed strictly on acetate. During growth on acetate, *Methanosarcina* species produce H_2_ (Lovley and Ferry, 1985), which is cycled back into the cells and reoxidized, with consecutive transfer of electrons and generation of proton motive force. H_2_ cycling is required for energy metabolism in *Methanosarcina* (Kulkarni et al., 2009). However, the H_2_ produced by *Methanosarcina* can be scavenged by hydrogenotrophic sulfate reducers (Phelps et al., 1985), parasitizing a key energy source for the methanogen. Here, Ozoulmes et al. show that H_2_ produced by a strict acetoclastic methanogen, *Methanosaeta concilii*, could also sustain the growth of sulfate reducers or hydrogenotrophic methanogens.

Zheng et al. observed that *Methanosarcina* spp. co-existed with *Geobacter* spp. in enrichments from iron-rich sediments of the Jiehe River provided with acetate. The authors suggested that *Geobacter* and *Methanosarcina mazei* perform syntrophic acetate oxidation in Jiehe's riverine sediments, which is possibly mediated by naturally abundant conductive minerals, such as magnetite.

Rotaru et al. reported on direct interspecies electron transfer (DIET) between *Methanosarcina barkeri* and different *Geobacter* species. The authors, learned that only high current density producing *Geobacter* species could interact via DIET syntrophy with *M. barkeri* (Rotaru et al.), whereas low current density producers did not. The authors suggested that greatest electrogens evolved effective EET with the purpose to interact syntrophically via DIET with other microorganisms, rather than minerals.

Moreover, a quinone-mediated interaction between *Geobacter* species (Liu et al., 2012) is described in depth by Smith et al. Quinone moieties are building blocks of humic acids-abundant constituents in soils and sediments. Smith et al. showed that a humic acid analog, AQDS, mediated electron transfer between two *Geobacter* species. When *Geobacter*-co-cultures were amended with AQDS, they did not need pili and cytochromes, components essential for DIET (Shrestha et al., 2013; Rotaru et al., 2014a,b).

## Mechanisms of extracellular electron transfer

The mechanisms employed by bacteria operating via EET are poorly understood. The best studied are *Geobacter* sp. and *Shewanella* sp. In *Geobacter* species, cell surface components (pili and type c cytochromes) are required for the organism to be able to grow using insoluble substrates (Lovley et al., 2011). However, in *Shewanella*, EET occurs via microbial exvaginations of cell membranes which carry redox-active molecules, e.g., multiheme cytochromes, responsible for charge transfer (Pirbadian et al., 2014).

Three papers in this topic studied EET in different organisms thereby expanding our understanding of EET mechanisms (Beckwith et al.; Dalla Vecchia et al.; Shi et al.).

Shi et al. screened all microbial genomes available, and learned that all *Geobacter* species and 11 phylogenetically and functionally diverse bacteria encoded a protein complex (Pcc) involved in trans-outermembrane electron transfer. The 11 new species spanned physiologies from Fe^3+^-reducers to anaerobic ammonium oxidizers. The authors suggested that the presence of the Pcc-cluster is an indication these organisms could use extracellular electron acceptors.

The study by Beckwith et al. investigated EET in *S. lithotrophicus*, an organism capable of Fe^2+^ oxidation. *S. lithotrophicus* employs membrane-bound cytochromes for electron uptake from Fe^2+^. The membrane-bound cytochromes of *S. litotrophicus* are encoded by *mtoAB*, resembling MtrA and MtrB from *Shewanella*. The exact mechanism of EET in *S. lithotrophicus* is unclear. Beckwith et al. anticipate that MtoD, a third c-type cytochrome co-transcribed with MtoAB, plays a role in EET. The authors provided a thorough biochemical and biophysical description of MtoD, and suggested that it plays a role in shuttling electrons between the outer-membrane bound MtoAB and inner-membrane bound electron acceptors.

Finally, EET mechanisms in Gram-positive organisms were studied in *Desulfotomaculum reducens* cultivated on insoluble iron-oxides (Dalla Vecchia et al.). EET in *D. reducens* is thought to be cytochrome-independent. The authors investigated cell surface proteins and discovered that only one redox-active protein, a homolog of alkyl hydroperoxide reductase, was differentially expressed in cells grown on iron oxides compared to cells grown fermentatively (Dalla Vecchia et al.). How this protein contributes to EET is not yet clear.

## Applications and engineering of EET

Two papers in this topic discussed the applicability of electroactive properties of microorganisms for sustainable manufacturing of chemicals. Tremblay and Zhang reviewed the use of electroautotrophic microorganisms as catalysts for the production of valuable chemicals when an electrode is the sole electron donor and CO_2_ is the only electron acceptor and carbon-source.

The second study revealed a novel bioengineering approach in engineered *Pseudomonas putida* (Schmitz et al.). The authors heterologously expressed a gene cluster for phenazine synthesis in the non-pathogenic *P. putida* KT2440, an organism of industrial interest, for the production of biodetergents. Usually, *P. putida* produced biodetergents under high-oxygen conditions, which prompts severe foaming. O_2_-limiting conditions can prevent foaming. Now, Schmitz et al. bioengineered *P. putida* to live under O_2_ limiting conditions with the electrode as electron acceptor, by using phenazine as EET shuttle. This study illustrated a path to O_2_-limited biocatalysis in *P. putida* and related organisms.

## Conclusion

EET has potential for future applications in sustainable technologies, but first we must learn how it works (Tremblay and Zhang). This research topic has collected recent groundbreaking advances in our understanding of what microbes utilize EET, how EET is carried out, and how it may be utilized in engineered systems. We hope that the topic persuades more researchers to explore EET in less investigated microorganisms, which are of interest for future applications.

## Author contributions

All authors listed, have made substantial, direct, and intellectual contribution to the work, and approved it for publication.

## Funding

AR would like to acknowledge the funding agencies which provided support for the duration of this research topic, namely two Natur of Univers, Det Frie Forskningsråd grants (an Independent research grant no. 1325-00022A, and a Sapere Aude Research Leadership Award no. 4181-00203), the Novo Nordisk Foundation, and Innovation fond no. 4106-00017B.

### Conflict of interest statement

The authors declare that the research was conducted in the absence of any commercial or financial relationships that could be construed as a potential conflict of interest.
